# Supplementary material for: Evidence for abundant transcription of non-coding regions in the Saccharomyces cerevisiae genome
Source: BMC Genomics. 2005 Jun 16;6:93. doi: 10.1186/1471-2164-6-93 (PMC1181813; doi:10.1186/1471-2164-6-93)
Supplement: Additional File 1 — Additional material. [file 1471-2164-6-93-S1.doc]

| YDR154C | 15571[2] | 13664[10] | 13419[10] | 12951[2] | 12214[10] |
| --- | --- | --- | --- | --- | --- |
| YHR217C | 8616[1] | 3215[1] | 2714[1] | 1185[1] | 1068[1] |
| YMR173W-A | 8520[10] | 6425[10] | 5637[10] | 5346[10] | 4998[10] |
| YDR133C | 7502[1] | 5259[9] | 5056[9] | 4869[10] | 4861[1] |
| YPR077C | 5766[1] | 4580[1] | 4329[1] | 4200[1] | 2461[1] |
| YOR309C | 4042[5] | 3180[5] | 2979[5] | 2766[12] | 2764[12] |
| YDR433W | 3591[10] | 3470[10] | 3233[5] | 3179[10] | 3098[10] |
| YLR294C | 3535[3] | 2444[6] | 2257[3] | 1962[10] | 1903[6] |
| YBR126W-A | 2733[6] | 2716[6] | 2367[4] | 2352[7] | 2307[6] |
| YOR203W | 2288[10] | 1985[10] | 1901[10] | 1670[12] | 1669[12] |
| YOR248W | 1727[8] | 1727[8] | 1727[8] | 1716[10] | 1716[10] |
| YER121W | 1478[1] | 1317[1] | 1241[1] | 1227[3] | 1193[3] |
| YNL017C | 1432[2] | 1004[2] | 806[2] | 272[2] | 214[2] |
| YEL033W | 1379[1] | 1175[2] | 1015[2] | 886[4] | 813[1] |
| YMR326C | 1286[1] | 452[1] | 270[3] | 194[3] | 126[1] |
| YGR182C | 1205[3] | 1155[3] | 855[1] | 837[1] | 808[1] |
| YIL100W | 1186[1] | 824[1] | 669[1] | 424[1] | 357[1] |
| YBR099C | 1181[10] | 752[10] | 677[10] | 631[9] | 567[10] |
| YGL188C | 1157[3] | 466[3] | 222[1] | 213[1] | 197[1] |
| YKL066W | 1152[3] | 910[4] | 834[3] | 808[1] | 632[1] |
| YPR053C | 1124[2] | 971[2] | 896[2] | 849[2] | 737[2] |
| YER084W | 1044[1] | 672[1] | 409[1] | 370[1] | 360[1] |
| YML089C | 1041[3] | 587[1] | 313[1] | 279[1] | 279[1] |
| YGR137W | 1024[10] | 884[10] | 833[10] | 654[9] | 643[10] |
| YLL020C | 1014[6] | 1011[6] | 971[6] | 826[6] | 793[10] |
| YOL085C | 985[1] | 379[1] | 278[1] | 256[1] | 234[3] |
| YBR206W | 973[3] | 776[1] | 578[2] | 572[10] | 564[3] |
| YKL225W | 940[10] | 570[10] | 409[9] | 339[9] | 331[10] |
| YHR214W-A | 866[1] | 790[1] | 768[1] | 656[1] | 616[1] |
| YEL014C | 813[10] | 196[10] | 152[8] | 152[8] | 152[8] |
| YKR040C | 806[9] | 266[10] | 157[10] | 109[10] | 81[12] |
| YBR116C | 770[6] | 651[6] | 633[1] | 597[3] | 581[1] |
| YNL184C | 699[3] | 609[3] | 481[3] | 379[3] | 326[3] |
| YDR544C | 694[1] | 510[1] | 413[1] | 267[1] | 259[3] |
| YKL123W | 688[3] | 505[3] | 473[10] | 430[1] | 370[10] |
| YPR146C | 682[2] | 600[2] | 395[2] | 391[2] | 345[2] |
| YDR401W | 681[8] | 57[1] | 52[1] | 43[1] | 40[7] |
| YBL048W | 675[7] | 607[6] | 576[6] | 553[1] | 489[1] |
| YOR015W | 660[3] | 517[1] | 506[12] | 506[12] | 506[12] |
| YFR020W | 659[6] | 570[6] | 567[6] | 541[6] | 464[3] |
| YDR504C | 653[2] | 574[10] | 568[3] | 554[6] | 507[2] |
| YNL043C | 651[10] | 612[1] | 472[1] | 470[1] | 266[1] |
| YOR013W | 642[2] | 429[2] | 353[1] | 321[10] | 298[2] |
| YBR012C | 626[4] | 414[10] | 264[2] | 248[1] | 234[1] |
| YLR184W | 609[10] | 409[10] | 382[12] | 381[12] | 357[12] |
| YGR151C | 602[8] | 596[4] | 570[3] | 549[8] | 548[12] |
| YMR103C | 600[1] | 515[6] | 490[10] | 434[10] | 432[10] |
| YLR261C | 594[10] | 448[3] | 400[10] | 366[10] | 361[10] |
| YDL158C | 588[4] | 294[8] | 283[8] | 274[6] | 266[8] |
| YLR252W | 578[10] | 558[3] | 552[1] | 506[10] | 422[1] |
| YPR044C | 572[2] | 257[2] | 227[12] | 216[2] | 210[11] |
| YLR202C | 566[7] | 317[7] | 273[7] | 213[10] | 184[10] |
| YOR072W | 563[1] | 399[2] | 361[1] | 324[1] | 319[1] |
| YER188W | 563[3] | 234[1] | 172[2] | 129[6] | 109[7] |
| YJR012C | 558[1] | 554[1] | 551[1] | 472[1] | 446[1] |
| YOR268C | 554[2] | 217[2] | 216[10] | 210[2] | 181[2] |
| YOR102W | 547[2] | 382[2] | 378[2] | 247[10] | 204[10] |
| YLR280C | 544[1] | 372[1] | 355[1] | 321[1] | 273[1] |
| YOR053W | 538[2] | 275[10] | 235[2] | 211[12] | 173[11] |
| YIL059C | 529[2] | 411[7] | 366[2] | 356[3] | 289[2] |
| YNL179C | 524[1] | 482[1] | 371[1] | 368[1] | 355[1] |
| YDR525W | 522[7] | 519[1] | 501[1] | 454[1] | 382[5] |
| YMR304C-A | 506[2] | 466[2] | 448[2] | 375[2] | 367[2] |
| YPL080C | 501[2] | 183[11] | 152[2] | 148[2] | 144[11] |
| YIL032C | 493[2] | 407[1] | 357[1] | 301[1] | 252[4] |
| YOR199W | 490[10] | 163[3] | 92[11] | 56[1] | 52[1] |
| YPR126C | 482[2] | 196[10] | 179[1] | 149[2] | 144[8] |
| YAL004W | 470[10] | 138[10] | 125[10] | 125[10] | 125[10] |
| YNR077C | 466[1] | 285[1] | 227[1] | 195[3] | 156[1] |
| YGL109W | 460[2] | 235[2] | 106[12] | 95[2] | 74[2] |
| YPL102C | 457[2] | 236[10] | 209[1] | 128[2] | 115[2] |
| YLR112W | 456[7] | 390[7] | 354[10] | 351[7] | 217[10] |
| YEL068C | 443[1] | 414[1] | 412[1] | 395[1] | 349[1] |
| YNL337W | 437[1] | 370[3] | 351[1] | 327[2] | 308[1] |
| YJL142C | 434[3] | 424[1] | 361[1] | 357[3] | 344[7] |
| YOR105W | 431[1] | 352[1] | 336[3] | 328[1] | 299[1] |
| YLR282C | 428[1] | 143[10] | 123[1] | 118[10] | 108[1] |
| YLL065W | 423[1] | 407[12] | 299[1] | 119[1] | 89[7] |
| YJR157W | 421[1] | 379[1] | 120[1] | 107[1] | 79[1] |
| YBL083C | 417[1] | 414[3] | 399[1] | 365[5] | 345[3] |
| YBL109W | 414[7] | 228[7] | 148[7] | 129[5] | 127[6] |
| YKL076C | 414[1] | 375[1] | 337[1] | 321[1] | 296[1] |
| YLR111W | 412[1] | 259[1] | 191[1] | 118[1] | 104[1] |
| YNL319W | 410[10] | 263[10] | 111[2] | 79[10] | 66[2] |
| YFL032W | 404[1] | 373[10] | 327[10] | 285[3] | 278[1] |
| YPR142C | 397[10] | 133[10] | 67[2] | 60[2] | 53[10] |
| YJL150W | 392[1] | 72[10] | 71[10] | 53[10] | 52[1] |
| YNL140C | 390[10] | 289[1] | 264[1] | 229[11] | 215[2] |
| YPR150W | 388[3] | 171[1] | 111[1] | 94[1] | 90[1] |
| YDR094W | 383[3] | 382[3] | 292[3] | 275[3] | 270[1] |
| YPL142C | 379[2] | 151[2] | 144[10] | 129[2] | 118[10] |
| YGL193C | 370[10] | 322[2] | 320[2] | 303[2] | 302[2] |
| YPL182C | 367[2] | 314[10] | 266[10] | 256[2] | 198[11] |
| YDR157W | 366[8] | 321[4] | 276[8] | 273[12] | 272[12] |
| YBR113W | 364[8] | 349[8] | 346[8] | 264[8] | 264[8] |
| YLR311C | 363[3] | 290[1] | 254[1] | 210[1] | 207[1] |
| YDL009C | 361[3] | 195[1] | 184[12] | 179[6] | 178[10] |
| YHL045W | 361[2] | 216[10] | 165[2] | 119[2] | 101[12] |
| YJL028W | 360[1] | 335[1] | 201[1] | 166[1] | 162[1] |
| YLR465C | 356[1] | 327[3] | 281[1] | 208[1] | 186[1] |
| YNL013C | 355[2] | 221[11] | 157[10] | 157[11] | 154[2] |
| YFL063W | 343[2] | 303[2] | 278[2] | 176[2] | 155[2] |
| YOR379C | 341[10] | 148[1] | 133[1] | 124[10] | 120[1] |
| YOR376W | 341[10] | 65[1] | 59[2] | 52[1] | 51[1] |
| YGR073C | 336[2] | 310[2] | 301[2] | 263[2] | 257[2] |
| YEL074W | 330[7] | 290[7] | 241[7] | 144[5] | 142[5] |
| YKL044W | 325[7] | 251[1] | 236[10] | 230[7] | 179[1] |
| YEL067C | 321[1] | 240[1] | 212[1] | 206[1] | 198[1] |
| YPL251W | 320[2] | 170[1] | 134[1] | 111[11] | 98[10] |
| YKR012C | 318[1] | 293[3] | 270[1] | 221[1] | 209[1] |
| YPR096C | 317[2] | 111[2] | 102[2] | 82[11] | 76[12] |
| YNL303W | 316[4] | 211[1] | 183[8] | 173[7] | 162[10] |
| YGR269W | 309[1] | 266[1] | 152[1] | 123[10] | 118[2] |
| YGR228W | 308[12] | 285[12] | 272[12] | 262[12] | 247[12] |
| YOR364W | 303[10] | 124[11] | 52[1] | 43[1] | 40[7] |
| YLR198C | 303[8] | 280[12] | 276[5] | 238[8] | 232[8] |
| YMR245W | 303[10] | 245[2] | 198[11] | 190[10] | 161[2] |
| YBR292C | 301[1] | 118[1] | 116[1] | 110[1] | 109[1] |
| YOL079W | 300[2] | 72[10] | 53[10] | 52[1] | 46[11] |
| YCR087W | 298[8] | 298[8] | 298[8] | 297[8] | 270[8] |
| YOL150C | 297[2] | 249[2] | 221[2] | 204[2] | 163[10] |
| YIL141W | 296[2] | 184[8] | 181[10] | 177[10] | 150[2] |
| YOR082C | 294[2] | 151[2] | 142[10] | 140[11] | 137[11] |
| YDR215C | 293[1] | 188[1] | 173[1] | 172[1] | 122[1] |
| YLR076C | 286[10] | 280[8] | 280[8] | 280[8] | 253[10] |
| YHR145C | 284[10] | 141[9] | 137[8] | 135[10] | 127[2] |
| YJR162C | 284[1] | 234[1] | 97[1] | 84[3] | 79[1] |
| YMR254C | 272[8] | 89[10] | 52[1] | 43[1] | 40[7] |
| YER066C-A | 267[10] | 127[2] | 120[2] | 99[11] | 82[10] |
| YKR035C | 266[10] | 177[10] | 156[8] | 139[8] | 139[8] |
| YCR013C | 261[3] | 228[1] | 225[10] | 158[3] | 142[10] |
| YDR209C | 261[6] | 206[6] | 149[6] | 147[7] | 147[6] |
| YJL199C | 261[6] | 241[7] | 227[6] | 221[6] | 209[10] |
| YCR041W | 258[3] | 62[3] | 54[10] | 53[10] | 52[1] |
| YGR122C-A | 257[6] | 229[6] | 218[6] | 154[6] | 138[6] |
| YNL285W | 257[1] | 256[1] | 241[1] | 205[1] | 173[1] |
| YNR042W | 254[2] | 227[2] | 220[2] | 209[10] | 174[2] |
| YBL096C | 252[8] | 249[8] | 211[8] | 192[4] | 184[10] |
| YMR290W-A | 252[2] | 130[11] | 107[10] | 95[2] | 91[11] |
| YGL199C | 251[2] | 237[2] | 213[12] | 153[12] | 145[10] |
| YLR317W | 250[7] | 195[7] | 187[7] | 135[5] | 129[10] |
| YPR123C | 249[2] | 186[7] | 176[2] | 133[2] | 98[10] |
| YNL170W | 238[1] | 206[2] | 196[10] | 191[1] | 169[11] |
| YGR011W | 232[2] | 220[3] | 171[1] | 163[4] | 162[3] |
| YGR039W | 230[10] | 76[6] | 52[1] | 50[6] | 43[1] |
| YDR543C | 230[1] | 209[3] | 204[10] | 193[12] | 190[3] |
| YOR135C | 229[2] | 116[2] | 113[2] | 98[10] | 83[1] |
| YKL147C | 229[1] | 140[1] | 89[10] | 88[1] | 81[1] |
| YMR316C-B | 228[2] | 128[2] | 115[2] | 92[11] | 88[2] |
| YPL197C | 227[2] | 130[11] | 126[10] | 123[10] | 87[2] |
| YPL238C | 226[2] | 200[4] | 182[4] | 181[4] | 169[4] |
| YLR349W | 225[10] | 166[1] | 134[6] | 129[1] | 127[10] |
| YLR123C | 224[1] | 157[10] | 127[10] | 102[10] | 97[11] |
| YDL023C | 223[1] | 157[1] | 144[10] | 144[1] | 123[10] |
| YBL053W | 222[8] | 204[8] | 199[10] | 188[8] | 172[8] |
| YNL150W | 222[2] | 201[2] | 194[2] | 145[12] | 144[10] |
| YDR537C | 221[1] | 210[1] | 140[1] | 124[1] | 94[1] |
| YOR169C | 220[12] | 163[12] | 148[12] | 148[12] | 134[12] |
| YKL169C | 217[6] | 157[6] | 138[6] | 127[6] | 110[12] |
| YGL260W | 217[10] | 87[1] | 64[1] | 40[7] | 39[7] |
| YJL067W | 216[10] | 143[10] | 143[10] | 104[2] | 91[7] |
| YAL045C | 214[10] | 123[10] | 120[8] | 71[10] | 71[10] |
| YPR136C | 213[2] | 83[2] | 52[1] | 51[10] | 46[11] |
| YOL106W | 210[2] | 159[2] | 156[2] | 133[10] | 124[2] |
| YDR220C | 204[10] | 169[8] | 157[10] | 134[8] | 133[8] |
| YLR279W | 204[10] | 141[12] | 138[10] | 125[10] | 125[10] |
| YPL044C | 204[10] | 59[7] | 52[1] | 43[1] | 40[7] |
| YNR025C | 203[2] | 189[2] | 180[2] | 121[2] | 120[1] |
| YPR012W | 202[2] | 196[10] | 180[2] | 134[12] | 134[12] |
| YGR160W | 200[10] | 169[10] | 132[10] | 127[12] | 106[12] |
| YGL204C | 198[10] | 172[2] | 169[2] | 157[2] | 146[4] |
| YAR069C | 196[10] | 112[7] | 95[8] | 90[10] | 84[7] |
| YKL115C | 193[4] | 117[4] | 114[7] | 111[4] | 107[4] |
| YNL198C | 191[1] | 177[10] | 99[2] | 99[11] | 98[2] |
| YER067C-A | 187[6] | 167[6] | 155[6] | 148[6] | 140[7] |
| YOR343C | 183[2] | 147[8] | 103[11] | 101[3] | 98[6] |
| YAL066W | 181[10] | 91[8] | 82[10] | 59[10] | 54[8] |
| YDL242W | 181[10] | 67[1] | 57[10] | 55[1] | 53[10] |
| YNL114C | 179[12] | 178[10] | 174[7] | 169[12] | 169[12] |
| YGL072C | 179[2] | 126[2] | 123[6] | 112[2] | 106[6] |
| YJL119C | 178[10] | 140[1] | 124[1] | 100[1] | 90[10] |
| YIR020C | 177[10] | 108[10] | 102[2] | 101[2] | 100[2] |
| YOR333C | 177[10] | 137[2] | 135[3] | 115[11] | 114[10] |
| YGL102C | 175[2] | 144[10] | 141[2] | 139[8] | 138[10] |
| YGL088W | 175[10] | 153[10] | 150[10] | 137[10] | 133[10] |
| YDL071C | 173[2] | 157[10] | 125[10] | 98[4] | 91[5] |
| YKR047W | 171[4] | 162[12] | 161[10] | 161[10] | 159[12] |
| YDL172C | 169[8] | 159[12] | 158[10] | 148[8] | 146[8] |
| YLR101C | 169[10] | 111[8] | 110[8] | 110[8] | 110[8] |
| YLR434C | 164[12] | 163[10] | 154[4] | 139[4] | 128[7] |
| YLR374C | 163[10] | 152[1] | 107[10] | 107[10] | 72[10] |
| YJL152W | 163[10] | 131[1] | 128[1] | 116[1] | 99[6] |
| YOR200W | 163[10] | 157[11] | 152[11] | 138[2] | 59[10] |
| YLR402W | 163[10] | 135[1] | 113[4] | 107[4] | 95[10] |
| YEL045C | 160[2] | 88[9] | 85[10] | 84[10] | 67[10] |
| YDL050C | 158[10] | 124[8] | 104[8] | 104[8] | 104[8] |
| YKL083W | 158[10] | 130[11] | 123[8] | 121[8] | 121[8] |
| YDR327W | 157[10] | 83[1] | 82[10] | 82[8] | 79[10] |
| YEL008W | 157[10] | 107[10] | 102[10] | 90[1] | 83[1] |
| YOR225W | 157[10] | 152[11] | 133[2] | 127[12] | 123[10] |
| YJL009W | 156[12] | 148[2] | 145[10] | 144[12] | 138[12] |
| YEL028W | 150[11] | 136[2] | 125[2] | 117[2] | 107[2] |
| YPR014C | 145[10] | 93[2] | 91[2] | 75[1] | 71[1] |

**Supplementary Table 1**: 164 strongly expressed DO along with their 5 highest expression values and their origin. Strongly expressed DO (SDO) are defined as dubious ORFs expressed above the 70^th^ percentile cutoff at least in one condition in our data set and have no overlap with non-dubious ORF. To get the expression values appear in the table each experiment was normalized using the procedure described in [1] (see also [http://expression.gnf.org/faq.html#scale](http://expression.gnf.org/faq.html#scale)): the lowest and highest 2% of the expression data were removed and the average expression (PM-MM) value of the remaining probes was tuned to 200. The origin of each value in the table appears in square parentheses beside the number. Use supplementary table 2 for the actual references. For each ORF the expression values are sorted from left to right. ORFs are sorted by highest expression value.

| (a) | (b) |
| --- | --- |
| 1 | [2] |
| 2 | [3] |
| 3 | [4] |
| 4 | [5] |
| 5 | [6] |
| 6 | [7] |
| 7 | [8] |
| 8 | [9] |
| 9 | [10] |
| 10 | [11] |
| 11 | [12] |
| 12 | [13] |

**Supplementary table 2**: References for supplementary table 1. (a) Reference # as appears in table 1. (b) Corresponding reference in the references list.

**Supplementary Table 3**: Comparison between PC distributions for S/AS DO-non-DO pairs and various sets of random pairs. The columns are: **(1)** Reference of the study left out. **(2)** # of remaining DO pairs with calculable PC, ( # of remaining S/AS SD. **(3)** Remaining # of conditions. **(4)** Average (Ave.) and standard deviation (SD) for the S/AS distribution. **(5)** Ave. and SD for random DO and non-DO pairs from the S/AS pairs (DO reshuffling). Here the random distribution was obtained using 103479 reshuffled pairs. **(6)** χ^2^ DF, and *P*, comparing S/AS and reshuffled distributions. **(7)** Ave. and SD for random pairs of non-DO from the S/AS set and random DO. **(8)** χ^2^ DF, and *P*, comparing S/AS and random SAS-no-DO/DO distributions. **(9)** Ave. and SD for random pairs of DO from the S/AS set and non-DO. **(10)** ^^ DF, and *P*, comparing S/AS and random SAS-DO/non-DO distributions. **(11)** Ave. and SD for random DO/non-DO pairs. **(12)** χ^2^of degrees of freedom (DF), and probability (*P*) comparing distributions of S/AS and random DO/non-DO pairs. All distributions but those of columns 4 and 5, were obtained using 33300 random pairs.

| (1) Exp. left out | (2) # pairs  (# strong  pairs) | (3) # cond. | (4) SAS  Ave.  SD | (5) DO-SAS/  non-DO-SAS | (6)  DF  χ^2^  *p* | (7) non-DO-SAS/DO | (8)  DF  χ^2^  *p* | (9) DO-SAS/ non-DO | (10)  DF  χ^2^  *p* | (11)  DO/  non-DO | (12)  DF  χ^2^  *p* |
| --- | --- | --- | --- | --- | --- | --- | --- | --- | --- | --- | --- |
| none | 333  (88) | 154 | 0.076  0.22 | -0.018  0.18 | 9  124  10^-22^ | -0.004  0.19 | 9  63  2×10^-10^ | -0.014  0.17 | 9  124  10^-12^ | 0  0.19 | 9  76  5×10^-13^ |
| [2] | 332  (83) | 130 | 0.102  0.22 | -0.017  0.18 | 9  197  7×10^-38^ | -0.003  0.19 | 9  123  2×10^-22^ | -0.01  0.18 | 9  200  2×10^-38^ | 0  0.19 | 10  132  9×10^-24^ |
| [3 ] | 332  (73) | 143 | 0.058  0.22 | -0.017  0.18 | 9  88  2×10^-15^ | -0.001  0.2 | 9  51  3×10^-8^ | -0.014  0.18 | 9  88  2×10^-15^ | 0.001  0.2 | 10  46  6×10^-7^ |
| [4] | 333  (86) | 146 | 0.08  0.22 | -0.015  0.18 | 9  122  2×10^-22^ | 0  0.19 | 9  66  4×10^-11^ | -0.01  0.18 | 9  112  3×10^-20^ | 0.003  0.19 | 9  67  3×10^-11^ |
| [5] | 331  (88) | 142 | 0.081  0.23 | -0.019  0.18 | 9  142  2×10^-16^ | -0.004  0.2 | 10  72  8×10^-12^ | -0.016  0.18 | 9  135  5×10^-25^ | 0  0.19 | 10  76  10^-12^ |
| [6] | 332  (88) | 143 | 0.072  0.22 | -0.021  0.18 | 9  108  2×10^-19^ | -0.005  0.2 | 9  56  3×10^-9^ | -0.017  0.18 | 9  106  4×10^-19^ | -0.002  0.2 | 9  63  2×10^-10^ |
| [7] | 332  (85) | 147 | 0.057  0.2 | -0.019  0.18 | 9  66  4×10^-11^ | -0.003  0.2 | 9  35  2×10^-5^ | -0.014  0.18 | 9  59  10^-9^ | 0.002  0.19 | 9  34  4×10^-5^ |
| [8] | 331  (87) | 151 | 0.074  0.22 | -0.02  0.18 | 9  172  1×10^-32^ | -0.006  0.2 | 9  88  2×10^-15^ | -0.017  0.18 | 9  220  1×10^-42^ | 0  0.19 | 9  86  5×10^-15^ |
| [9] | 333  (87) | 142 | 0.077  0.21 | -0.015  0.17 | 9  118  2×10^-21^ | -0.004  0.19 | 9  70  7×10^-12^ | -0.011  0.17 | 9  127  2×10^-23^ | 0.001  0.19 | 10  65  2×10^-10^ |
| [10] | 333  (88) | 150 | 0.073  0.22 | -0.019  0.18 | 9  122  2×10^-22^ | -0.005  0.19 | 9  66  4×10^-11^ | -0.013  0.18 | 9  114  10^-20^ | 0.001  0.19 | 10  63  4×10^-10^ |
| [11] | 326  (66) | 112 | 0.076  0.25 | -0.027  0.21 | 9  147  2×10^-27^ | -0.012  0.22 | 9  77  3×10^-13^ | -0.02  0.21 | 9  125  6×10^-23^ | -0.008  0.21 | 9  90  8×10^-16^ |
| [12] | 333  (88) | 150 | 0.074  0.22 | -0.019  0.18 | 9  122  2×10^-22^ | -0.004  0.19 | 9  66  4×10^-11^ | -0.013  0.18 | 9  115  7×10^-21^ | 0  0.19 | 9  65  6×10^-11^ |
| [13] | 328  (87) | 138 | 0.076  0.22 | -0.018  0.18 | 9  125  6×10^-23^ | -0.004  0.19 | 9  72  3×10^-12^ | -0.014  0.18 | 9  136  3×10^-25^ | 0.002  0.19 | 9  74  10^-12^ |

Reference list

1. Su AI, Cooke MP, Ching KA, Hakak Y, Walker JR, Wiltshire T, Orth AP, Vega RG, Sapinoso LM, Moqrich A *et al*: **Large-scale analysis of the human and mouse transcriptomes**. *Proc Natl Acad Sci U S A* 2002, **99**(7):4465-4470.

2. Primig M, Williams RM, Winzeler EA, Tevzadze GG, Conway AR, Hwang SY, Davis RW, Esposito RE: **The core meiotic transcriptome in budding yeasts**. *Nat Genet* 2000, **26**(4):415-423.

3. Cohen BA, Pilpel Y, Mitra RD, Church GM: **Discrimination between paralogs using microarray analysis: application to the Yap1p and Yap2p transcriptional networks**. *Mol Biol Cell* 2002, **13**(5):1608-1614.

4. Williams RM, Primig M, Washburn BK, Winzeler EA, Bellis M, Sarrauste de Menthiere C, Davis RW, Esposito RE: **The Ume6 regulon coordinates metabolic and meiotic gene expression in yeast**. *Proc Natl Acad Sci U S A* 2002, **99**(21):13431-13436.

5. Bernstein BE, Tong JK, Schreiber SL: **Genomewide studies of histone deacetylase function in yeast**. *Proc Natl Acad Sci U S A* 2000, **97**(25):13708-13713.

6. Bulik DA, Olczak M, Lucero HA, Osmond BC, Robbins PW, Specht CA: **Chitin synthesis in Saccharomyces cerevisiae in response to supplementation of growth medium with glucosamine and cell wall stress**. *Eukaryot Cell* 2003, **2**(5):886-900.

7. Bro C, Regenberg B, Lagniel G, Labarre J, Montero-Lomeli M, Nielsen J: **Transcriptional, proteomic, and metabolic responses to lithium in galactose-grown yeast cells**. *J Biol Chem* 2003, **278**(34):32141-32149.

8. Sabet N, Tong F, Madigan JP, Volo S, Smith MM, Morse RH: **Global and specific transcriptional repression by the histone H3 amino terminus in yeast**. *Proc Natl Acad Sci U S A* 2003, **100**(7):4084-4089.

9. Robertson LS, Causton HC, Young RA, Fink GR: **The yeast A kinases differentially regulate iron uptake and respiratory function**. *Proc Natl Acad Sci U S A* 2000, **97**(11):5984-5988.

10. Kobor MS, Archambault J, Lester W, Holstege FC, Gileadi O, Jansma DB, Jennings EG, Kouyoumdjian F, Davidson AR, Young RA *et al*: **An unusual eukaryotic protein phosphatase required for transcription by RNA polymerase II and CTD dephosphorylation in S. cerevisiae**. *Mol Cell* 1999, **4**(1):55-62.

11. Holstege FC, Jennings EG, Wyrick JJ, Lee TI, Hengartner CJ, Green MR, Golub TR, Lander ES, Young RA: **Dissecting the regulatory circuitry of a eukaryotic genome**. *Cell* 1998, **95**(5):717-728.

12. Geisberg JV, Holstege FC, Young RA, Struhl K: **Yeast NC2 associates with the RNA polymerase II preinitiation complex and selectively affects transcription in vivo**. *Mol Cell Biol* 2001, **21**(8):2736-2742.

13. Wyrick JJ, Holstege FC, Jennings EG, Causton HC, Shore D, Grunstein M, Lander ES, Young RA: **Chromosomal landscape of nucleosome-dependent gene expression and silencing in yeast**. *Nature* 1999, **402**(6760):418-421.
